# Dedicated mobile application for drug adverse reaction reporting by patients with relapsing remitting multiple sclerosis (Vigip-SEP study): study protocol for a randomized controlled trial

**DOI:** 10.1186/s13063-018-2560-4

**Published:** 2018-03-09

**Authors:** Gilles Defer, Florian Le Caignec, Sophie Fedrizzi, François Montastruc, Damien Chevanne, Jean-Jacques Parienti, Laure Peyro-Saint-Paul

**Affiliations:** 1 Neurology Department, CHU de Caen, 14000 Caen, France; 2 Clinical Research Department, CHU de Caen, 14000 Caen, France; 3 Pharmacology Department, CHU de Caen, 14000 Caen, France; 4 Pharmacology Department, CHU de Toulouse, 31000 Toulouse, France

**Keywords:** Pharmacovigilance, E-health, Patient reporting, Multiple sclerosis, Adverse drug reaction, Mobile application

## Abstract

**Background:**

The reporting of adverse drug reactions (ADR) by patients represents an interesting challenge in the field of pharmacovigilance, but the reporting system is not adequately implemented in France. In 2015, only 20 MS patients in France reported ADR due to first-line disease-modifying drugs (DMD), while more than 3000 patients were initiated on DMD.

The aim of this study is to validate a proof-of-concept as to whether the use of a mobile application (App) increases ADR reporting among patients with relapsing-remitting multiple sclerosis (RR-MS) receiving DMD.

**Methods/design:**

We designed a multi-centric, open cluster-randomized controlled trial, called the Vigip-SEP study (NCT03029897), using the App My eReport France® to report ADR to the appropriate authorities in E2B language, in accordance with European regulations. RR-MS patients who were initiated on, or switched, first-line DMD will be included. In the experimental arm, a neurologist will introduce the patient to the App to report ADR to the appropriate French authorities. In the control arm, the patient will be informed of the existence of the App but will not be introduced to its use and will then report ADR according to the usual reporting procedures. Primary assessment criteria are defined as the average number of ADR per patient and per center. We assume that the App will increase patient reporting by 10-fold. Therefore, we will require 24 centers (12 per arm: 6 MS academic expert centers, 3 general hospitals, 3 private practice neurologists), allowing for an expected enrollment of 180 patients (alpha risk 5%, power 90% and standard deviation 4%).

**Discussion:**

Increasing patient reporting of ADR in a real-life setting is extremely important for therapeutic management of RR-MS, particularly for monitoring newly approved DMD to gain better knowledge of their safety profiles. To increase patient involvement, teaching patients to use tools, such as mobile applications, should be encouraged, and these tools should be tested rigorously.

**Trial registration:**

ClinicalTrials.gov, ID: NCT03029897. Registered on 20 January 2017.

**Electronic supplementary material:**

The online version of this article (10.1186/s13063-018-2560-4) contains supplementary material, which is available to authorized users.

## Background

Adverse drug reactions (ADR) constitute some of the major causes of morbidity and mortality worldwide. It is estimated that ADR account for more than 5% of hospital admissions, 28% of emergency department visits and 5% of deaths during hospitalization [1]. Thus, ADR caused approximately 200,000 deaths per year in Europe and generated costs of 79 million euros in 2015 alone [2].

Spontaneous reporting of ADR is the backbone of the pharmacovigilance system and has been proven to play a role in identifying signs related to drug safety. Descriptions of the safety profiles of drugs during clinical development are not exhaustive and do not reflect potentially late or rare occurrences of ADR [1]. Spontaneous reporting by health care professionals in subsequent post-marketing settings is the greatest source of drug safety data [3]. However, under-reporting of ADR is a key limitation of the efficiency of pharmacovigilance systems, and spontaneous reporting by patients is now recognized as a new source of information for these systems.

A recent review of the academic literature confirmed that patient reporting adds new information about ADR: such reporting provides a more detailed description of ADR that differs in terms of the nature of the reaction and the suspected drugs involved [4]. In the Netherlands from 2010 to 2015, patients made important contributions by reporting 26% of all ADR reports [3, 5]. The Danish Medicines Agency published a report on ADR reported by consumers compared with reports from health care professionals covering the period from 2003 to 2011 and showed that consumer reports contribute significantly to the total number of ADR reports, both quantitatively and qualitatively [6]. Patient self-reporting was also investigated in the United Kingdom (UK) in the UK’s Yellow Card Scheme from 2005 to 2007, and the results also suggested that patient reporting provides a positive complementary contribution to health professional reporting [7, 8]. In France, during the pandemic influenza vaccination campaign in 2009–2010, a pharmacovigilance plan was introduced following the recommendations of the European Medicines Agency (EMA). For the first time in France, patients were able to report ADR concerning the pandemic vaccine directly to pharmacovigilance centers. This study revealed no major qualitative differences between patients’ and health professionals’ reports [9]. Despite growing interest and acceptance of ADR reporting by patients [10, 11], few studies have validated the relevance of data from patient notifications [12–14].

Health authorities in 44 countries currently emphasize spontaneous ADR notifications by patients and the need for a more accessible reporting system [15]. According to new European legislation (Directive 2010/84/EU), member states are required to establish an ADR reporting system for patients. Since 2011, patients in France have been able to self-report their ADR without involving a health professional. The French pharmacovigilance system is based on 31 regional drug monitoring centers and a dedicated unit of the French Agency “Agence nationale de la sécurité du médicament et des produits de santé (ANSM).” Patients can report ADR either directly to ANSM via a form that is available at http://ansm.sante.fr/ or to a regional center electronically or by telephone. However, patient reporting in France remains underdeveloped, and its availability is not widely known by the general population [16]. For example, in 2014, ADR reported by patients represented 4.5% of all reports, compared with 10% in Europe, according to data from the French National Database of Pharmacovigilance [16]. Most patients remain unaware of reporting systems or are confused about reporting [17].

Therefore, “e-health” applications could be a convenient method to more closely connect with patients [18]. It has been demonstrated that the use of mobile applications to report adverse events, such as those related to a medical device, can save time and increase the number of notifications. On average, there were 15 times more reports submitted per month via the App MedWatch® with patient community support than submitted via traditional pharmacovigilance portals [19].

In 2016, it was estimated that 50% of the population and 99% of young physicians in France own a smartphone. The most widely used medical Apps by young physicians are Apps related to drugs (drug database, drug interaction database) [20]. A smartphone/tablet application seems to be a necessity for modern pharmacovigilance approaches (i.e., MedWatcher® in USA or YellowCArd® in UK).

In France, several applications dedicated to ADR reporting are available on the market (i.e., VigiBip®, My e-Report®, Medisafe®), but these applications are not yet widely used by patients. For example, in the Toulouse University Pharmacovigilance Center, patient reports submitted by the App VigiBip® (6.7% of total reports received by the center) were significantly more frequent than those submitted via classical methods (3.4%) (*p* = 0.01) [21].

To further promote patient involvement, teaching tools, such as mobile applications, should be encouraged, and these tools should be tested rigorously.

### Population

During the past years, different new disease-modifying drugs (DMD) have become available on the market for multiple sclerosis (MS) care, although most of these drugs induce significant general and biological side effects. While these new treatments offer important medical benefits, they also require close monitoring, which has led to the implementation of risk management plans (RMP). Clinical trial populations are homogenous and rigorously followed, whereas under-reporting by patients is undeniable in normal clinical practice.

Hence, increasing the rate of spontaneous reports of ADR by MS patients would represent a significant contribution to the pharmacovigilance system. MS occurs in young adults, which is a population that is accustomed to the use of smartphones and Apps. Therefore, utilizing an App to report ADR could stimulate spontaneous reporting by patients.

According to data extracted on 15 December 2016 from the French MS Observatory “Observatoire Français de la Sclérose en Plaques (OFSEP)” based on an analysis of the French patient cohort EDMUS (http://www.ofsep.org/fr/la-cohorte-ofsep/descriptif-de-la-cohorte), each year, 5000 new patients are diagnosed with MS in France. The distribution of the various forms of MS is as follows: 79% relapsing-remitting multiple sclerosis (RR-MS), 11% progressive MS and 10% a clinically isolated syndrome. This trial will focus on patients with RR-MS, as these patients are involved in the management and monitoring of their pathology, with a population of 3950 newly diagnosed individuals per year in France. Furthermore, we know that 25% of RR-MS patients do not receive DMD (extracted from OFSEP). Thus, approximately 3000 first-line DMD evaluated in RR-MS patients are introduced per year in France. First-line DMD include interferon β, peginterferon β, glatiramer acetate, teriflunomide, and dimethyl fumarate.

#### Intervention

All patients included in Vigip-SEP will be informed that they can report their ADR using a pharmacovigilance system. They will also be informed of the existence of the App My eReport France® developed to report ADR in accordance with European and French regulations. In experimental centers, patients will be introduced to its use by neurologists. In control centers, they will not be introduced to its use but will be encouraged to report ADR through usual reporting methods.

The My eReport France® application was chosen for the Vigip-SEP study, developed by the eVeDrug® society, to report ADR to the appropriate French authorities in E2B language, in accordance with European regulations. The application is certified by the label “My health quality.” Data hosting is provided by a certified health data host. Updates are performed automatically according to the evolution of E2B regulations.

#### Comparison

The mean number of ADR per patient at the center will be compared between interventional and control centers.

#### Outcome

The principal aim of the Vigip-SEP study is to validate a proof-of-concept: Does the use of a mobile application (App) increase ADR reporting in RR-MS patients receiving first-line DMD?

## Methods/design

### Aim, design and setting

The overall aim of our Vigip-SEP trial is to study reporting of ADR by RR-MS patients using a mobile application.

The first aim is to evaluate the increase in ADR reporting by mobile application use. The second aim is to evaluate the quality of patient reporting. We will also evaluate the effect of patient involvement in pharmacovigilance on physician reporting (boost effect). Additionally, the user-friendliness of the application will be assessed according to the patients’ satisfaction, the patients’ judgment of practicability, and the patients’ suggestions for improvements.

The designed study is a multi-centric, open cluster-randomized controlled trial.

The Vigip-SEP study is set in France. The trial sponsor is CHU de Caen, an academic hospital, responsible for protocol decisions and quality control. Coordination is provided by an investigative team of MS academic experts at the center of CHU de Caen.

This study was approved by the Ethics Committee of Nord-Ouest III with approval number 2016–42. The privacy of the participants and their personal medical records will be guaranteed by treating the data according to the French law n. 78–17 of 6 January 1978 and the “European Union Data Protection Directive (95/46/EC 24 October 1995).”

The approved version of Vigip-SEP is V3 29.11.2016; the recruitment began in June 2017, and the end date is anticipated in May 2018.

The sponsor CHU de Caen is responsible for reporting any protocol modifications to the centers, ethic committees and ANSM.

### Subjects

To be eligible for participation, subjects must meet the following defined inclusion criteria:

- Men or women aged ≥ 18 years with RR-MS
- Patients undergoing the initiation of a first-line DMD (interferon β, peginterferon β, glatiramer acetate, teriflunomide, and dimethyl fumarate). Patients without ulterior first-line DMD or patients benefiting from a change in treatment between first-line DMD (switch)
- Patients who have access to a smartphone, tablet or other computer device that can host the selected mobile application
- Patients benefiting from the French Social Security system
- Satisfactory level of comprehension and expression in French
- Patients who provided written informed consent

Subjects meeting any one of the following exclusion criteria may not be eligible to participate in the study:

- Patients with secondary or primary progressive MS
- Patients with MS who are not being treated with a first-line DMD

### Description of all processes (see Fig. 1)

#### Randomization

The biostatistician of the sponsor CHU de Caen will perform the randomization. Randomization will be performed using the ALEA function of Microsoft Excel 2013.

**Fig. 1:**
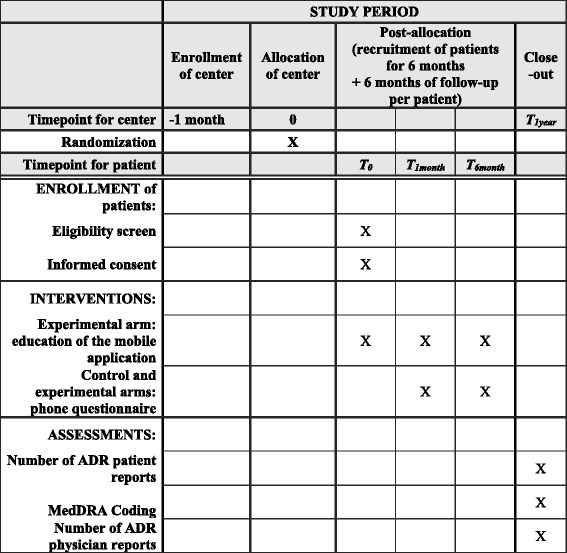
Enrollment, interventions and assessments in the Vigip-SEP study

Centers will be stratified based on structure type then randomly allocated (1:1) to the experimental arm (these centers will introduce all patients to use of the App) or to the control arm (these centers will not introduce patients to use of the App). Centers will be informed of the randomization the day of the trial initiation visit.

Clustering avoids a “contamination” bias (in the epidemiological sense) of different patients followed by the same prescriber.

Neurologists will successively include all patients meeting the criteria until they have reached the number of subjects to be included. In 1 year, MS academic expert centers are expected to include 10 patients, and general hospitals and private practice neurologists are expected to include 5 patients. Neurologists will inform patients about the Vigip-SEP study and obtain the patients’ written informed consent.

CHU de Caen conforms to National Informatic and Liberty French Law and, therefore, patient confidentiality will be protected.

#### Intervention arm

In the experimental arm, the patient is introduced to use of the App by a neurologist.

A video tutorial created by the investigator’s staff of the Vigip-SEP study is presented to the patient.

The patient is able to report his ADR via the mobile application. The report is electronically sent directly to the regional pharmacovigilance center, which assesses the case, and after treatment, records it in the national competent authority database in the usual manner.

Patients can also describe their ADR to their physician during consultation. A physician who is informed of an ADR by his patient is able to report it as usual (via the mobile application or via the conventional methods of reporting), and duplicate reports will be identified a posteriori.

A follow-up questionnaire will be administered to patients by telephone call during the study at 1 month (± 15 days). The patient will be asked if they had ADR and, if so, whether they reported the ADR and how. Education regarding the application will be monitored, and the user-friendliness of the application will also be evaluated by the patients’ satisfaction, practicability and suggestions.

#### Control arm

In the control arm, no intervention is performed except for follow-up questionnaires at 1 and 6 months. The patient is informed of the existence of the App but is not introduced to its use. The patient reports ADR according to the usual reporting procedures.

In both arms, any concomitant care is allowed. Usual care will be conducted in the post-trial period.

### Outcome measurements

#### Primary outcomes

The primary outcome is the number of ADR patient reports. ADR reports will be collected in both arms for 6 months after the inclusion of each patient from the national database of French authority, ANSM (see “Data collection procedure” below).

An example of the method of aggregation is as follows. For center 1, *m*1 = *n*1 reports/*N*1 patients, where *n*1 is the number of ADR in the center, and *N*1 is the number of patients included in the center.

The method of aggregation in each group follows the formula Ʃ*mx*/*N*, where *N* is the number of centers in the group.

#### Secondary outcomes

Each reaction is assessed by a regional pharmacovigilance center with a MedDRA coding. The profile of the ADR per drug classified by System Organ Class will be compared with the safety post-marketing profile in the Summary of Product Characteristics.

The number of physician reports will be compared between the two arms to measure the “boost” effect of patient reporting via the mobile application.

The user-friendliness of the application will be evaluated by analyzing the responses to the questionnaire completed at 1 month (± 15 days).

The feedback from the regional pharmacovigilance center will be evaluated by analyzing the responses to the questionnaire completed at 6 months (± 15 days).

#### Data collection procedure

The data manager of CHU de Caen is responsible for form development, sponsor database development and data management.

Regardless of how ADR are reported, they are sent to a regional pharmacovigilance center for assessment, which includes MedDRA coding and causality analysis. Then, the regional pharmacovigilance center records the ADR in the national database.

At the end of the study, the 31 regional centers of pharmacovigilance will anonymously extract ADR reports pertaining to the first-line DMD in the trial period from the national database. The investigator’s team will identify reports of the Vigip-SEP population (of the two groups), and the Vigip-SEP investigators will compare the received date and the region, age and sex of the patient. ADR reported by patients within the 6 months of inclusion and by neurologists within the same period will be recorded in the sponsor database. Duplicates (the same ADR reports made by patients and physicians) will then be identified.

Data collected regarding the investigating centers will include the following:

- Type of structure: MS academic expert center, general hospital, private practice neurologist
- Date and result of randomization
- Date of implementation
- Regional pharmacovigilance center attached to the investigating center

Data collected regarding patients will include the following:

- Socio-demographic information: patient initials, date of birth and gender
- Confirmation of diagnosis of RR-MS and date of diagnosis
- EDSS (Expanded Disability Status Scale) score at the time of inclusion
- Date of inclusion
- Name and date of initiation of first-line DMD prescribed
- Name of previous first-line DMD if the patient is not naïve of any MS treatment
- If a switch occurred in MS treatments during the course of the study, date of the current first-line DMD discontinuation and name and date of initiation of the new first-line DMD
- Adverse events: method of reporting, description, date, first-line DMD concerned, MedDra code, and expectedness (a previously known adverse reaction and its frequency, listed in the summary of the product characteristics of the drug)
- Responses to the questionnaire at 1 month (± 15 days), ADR, method of reporting, and friendliness of App (only in the experimental arm)
- Response to the questionnaire at 6 months (± 15 days) regarding feedback from the regional pharmacovigilance center

### Data analysis

The statistical analysis will be carried out by the Vigip-SEP scientific team.

#### Hypothesis

In the academic literature, no study has measured the impact of an App on increasing reports of ADR by patients. MedWatcher® is a user-friendly reporting App used to prepare and submit an adverse event report to the Food and Drug Administration for a hysteroscopic sterilization device. In 2012, a patient social media community adopted MedWatcher® to declare adverse reactions related to the device. On average, there were 15 times more reports via the App than via the traditional pharmacovigilance portal [19]. We assume that patient reporting will increase by 10-fold with the use of the mobile application My e-Report® over the traditional reporting method.

To follow the cluster-randomized design, the statistical unit will be the center. Therefore, the average number of ADR patient reports per patient will be pooled by center, and the comparison will be performed at the cluster (center) level. Estimation of number of centers required: this is a comparison of two means with an average for the arm standard of 20/3000 = 0.0067 or 6.7 per 1000 patients; and for the interventional arm of 200/3000 = 0.067 or 67 per 1000 patients. The power is 90% according to a bilateral test with 5% error risk and an estimated standard deviation of 0.04. Therefore, 10 centers in each arm are needed to test our hypothesis.

We will target to include 12 centers per arm to counter the trend in over-reporting due to informing physicians of the study (inflation factor). A total of 24 MS outpatient-clinic departments will recruit 180 patients (90 per arm) with the following distribution according to structure type: 10 patients per MS academic expert center, 5 patients per general hospital and 5 patients per private practice neurologist.

Statistical criteria for stopping the study: no intermediate analysis is planned, and the study will be stopped when all centers have obtained the required number of patients.

Method for accounting for missing, unused or invalid data: the patient is first informed of the study, and then, the patient’s statements are collected for 6 months. Each patient will be contacted by telephone 1 month (± 15 days) after inclusion. The 1-month follow-up questionnaire will be used to determine whether the patient should be removed from the study and replaced according to the following considerations:

- If the patient has reported at least one ADR, the patient data are preserved
- If the patient did not report an ADR:
  - Because they had no ADR, the patient data are preserved
  - Because they did not like the method of reporting, the patient data are preserved
  - For technical reasons (loss or breakage of the telephone/tablet), the patient is replaced by another patient, and these data are not analyzed

The sponsor CHU de Caen applies random audits of their trials of low-risk studies. The experimental intervention, defined as “Introduction to the App,” is not a risk for patients included in Vigip-SEP, and therefore, a safety monitoring board was not necessary.

There is no plan to provide access to the final data to anyone beyond the investigators.

#### Statistical analysis plan

A biostatistician will compare the mean number of ADR per patient at the center level by using Student’s *t* test or Wilcoxon’s rank test, as appropriate, with the number of enrolled patients in each center as a weighting factor. In addition, the biostatistician will generate hierarchical models (level 1: centers; level 2: physicians nested within centers; level 3: individuals nested within physicians) with an analysis at the level of the patient taking into account the intra-cluster correlations at different levels using a generalized linear model. This analysis will have the advantage of controlling for potential confounders at the individual and physician level. The results will be reported using the Consolidated Standards of Reporting Trials (CONSORT) criteria (see Additional file 1).

We further plan to communicate these results to pharmacologist and neurologist review and to congress.

## Discussion

Application use by patients could be an integral part of the pharmacovigilance system that is in place in France. This study aims to improve the efficiency of the pharmacovigilance system by increasing the number of reports and, therefore, allowing earlier detection of events to prevent and minimize risks.

The robust methodology, with a clustered randomized national study, will determine the usefulness of national support for reporting by mobile application.

The results of this study should be available in the summer of 2018, and we hope that these results will be consistent with our assumption that (1) the efficiency of the post-marketing authorization system will be improved by an increase in reporting of ADR via the mobile application and (2) the therapeutic safety of MS patients will be improved by a better knowledge of the safety profile of DMD.

Developing a culture of reporting ADR by RR-MS patients is a relevant goal. In 2015, in France, 20 ADR were reported by MS patients compared with 271 ADR reported by physicians concerning first-line DMD according to data from the French National Database of Pharmacovigilance. In the future, we hope that this new method of reporting will improve pharmacovigilance by RR-MS patients and can be extended to include all new DMD. The overall goal is to be able to improve the early detection of unexpected adverse reactions not revealed by clinical consultations. If our hypothesis is confirmed, other neurological diseases could be explored using this method.

### Strengths and limitations

This study has several strengths. First, the design, a randomized control trial, will allow a causal interpretation in terms of outcomes. In addition, including several types of centers (academic expert centers, general hospitals and private practice neurologists) will increase the external validity of the results. Furthermore, the study is focused on the use of an innovative tool, a mobile application, by a population in real-life conditions.

We are also aware of several potential limitations. This is an open trial and, therefore, over-reporting due to informing the patients and physicians is expected and cannot be neutralized. Moreover, patients failing to report ADR by the App may have had a technical problem or may have forgotten to report the ADR. Indeed, the user-friendliness of the mobile application has not been tested. To counter this limitation, 1 month after inclusion, we will ensure by a telephone call that patients report all ADR and are able to use the mobile application effectively. Feedback from regional pharmacovigilance centers will also be investigated by a telephone call at 6 months after inclusion to explore patient satisfaction [16].

Currently, patients describe ADR via the App via free verbatim that is later coded by pharmacologists of pharmacovigilance centers using the MedDra dictionary. Encouraging this reporting requires the accompaniment of regional pharmacovigilance centers. In addition, informing patients, as proposed in the study, requires time by neurologists. Financial resources should be put in place for this support, which could be included, for example, in a patient education program.

Pharmacovigilance is a major public health issue. First-line DMD, whose risk profiles are not fully known, that have been newly released to the market must be carefully monitored. Patient involvement in the pharmacovigilance system is already accepted to be a valuable asset but must be further developed. RR-MS patients, who are already well-adapted to managing their disease, could be key players in effective pharmacovigilance. In a second phase, we plan to develop applications specifically dedicated to new DMD.

### Trial status

Recruitment has not been completed at the time of this submission.

## Additional file

Additional file 1: SPIRIT 2013 Checklist: recommended items to address in a clinical trial protocol and related documents*. (DOCX 49 kb)

## Abbreviations

ADR: Adverse drug reactions
App: Mobile application
DMD: Disease-modifying drugs
E2B: E2B does not have a direct translation to English. The International Conference on Harmonization of Technical Requirements for Registration of Pharmaceuticals for Human Use (ICH) published guidelines that designated “E” to stand for efficacy. E2B essentially defines what data elements need to be transmitted in individual case safety reports (ICSRs), regardless of the source or destination
EMA: European Medicines Agency
MS: Multiple sclerosis
RMP: Risk management plans
RR-MS: Relapsing-remitting multiple sclerosis
WHO: World Health Organization
